# Current Status on Therapeutic Molecules Targeting Siglec Receptors

**DOI:** 10.3390/cells9122691

**Published:** 2020-12-15

**Authors:** María Pia Lenza, Unai Atxabal, Iker Oyenarte, Jesús Jiménez-Barbero, June Ereño-Orbea

**Affiliations:** 1 Chemical Glycobiology Laboratory, CIC bioGUNE, Basque Research and Technology Alliance, BRTA, Bizkaia Technology Park, Building 800, 48160 Derio, Spain; mlenza@cicbiogune.es (M.P.L.); uatxabal@cicbiogune.es (U.A.); ioyenarte@cicbiogune.es (I.O.); 2 Ikerbasque, Basque Foundation for Science, 48013 Bilbao, Spain

**Keywords:** Siglec, sialic acid, glycan, antibody, CAR

## Abstract

The sialic acid-binding immunoglobulin-type of lectins (Siglecs) are receptors that recognize sialic acid-containing glycans. In the majority of the cases, Siglecs are expressed on immune cells and play a critical role in regulating immune cell signaling. Over the years, it has been shown that the sialic acid-Siglec axis participates in immunological homeostasis, and that any imbalance can trigger different pathologies, such as autoimmune diseases or cancer. For all this, different therapeutics have been developed that bind to Siglecs, either based on antibodies or being smaller molecules. In this review, we briefly introduce the Siglec family and we compile a description of glycan-based molecules and antibody-based therapies (including CAR-T and bispecific antibodies) that have been designed to therapeutically targeting Siglecs.

## 1. General Introduction to Siglecs

**Figure 1:**
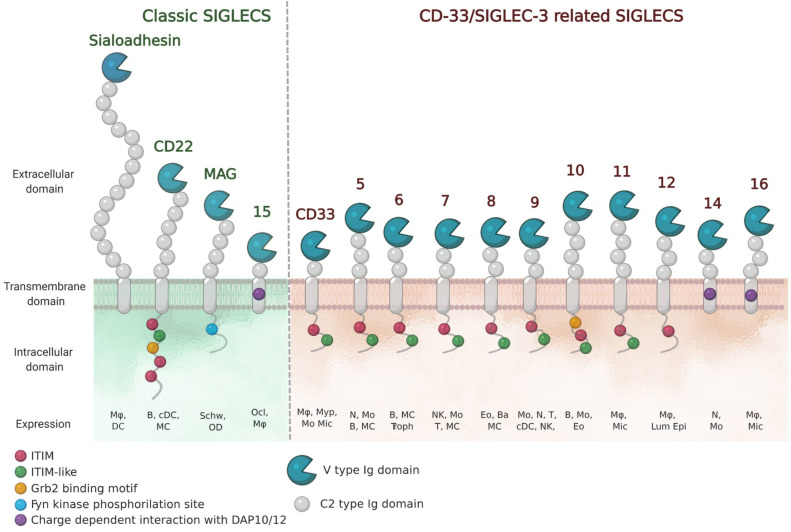
Schematic representation of human Siglec receptors. Siglecs contain one N-terminal V-type Ig-like domain that mediates sialic-acid recognition and varying numbers of constant (C)-type Ig-like domains at the extracellular domain. Siglecs can be divided into two groups (classic and CD33/Siglec-3 related) based on sequence similarity and evolutionary conservation. Siglec-13 is present in baboons and chimpanzees and is specifically deleted in humans. Siglec-12 in humans has lost the ability to bind sialic acids. The cell-expression patterns are shown (Mø, macrophages; DC, dendritic cell; B, B cells; MC, mast cells; Schw, Schwann cells; OD, oligodendrocytes; Ocl, osteoclasts; Myp, myeloid progenitor; Mo, monocytes; Mic, microglia; N, neutrophils; Troph, trophoblasts; NK, natural-killer cells; T, T cells; Eo, eosinophils; Ba, basophils; Lum epi, lumen epithelia cells).

The sialic acid binding immunoglobulin (Ig)-like lectins (Siglecs) family in humans is composed by 15 members and in general, they are all expressed in immune cells (Figure 1) [1,2]. The exceptions are myelin-associated glycoprotein (MAG) (or Siglec-4) that is expressed on oligodendrocytes and Schwann cells, and Siglec-6 on placental trophoblasts. Based on sequence conservation and evolution, Siglecs are divided in two subgroups: (i) classic Siglecs (including Sialoadhesin (Siglec-1), CD22 (Siglec-2), MAG and Siglec-15); and (ii) CD33-related Siglecs (CD33 (Siglec-3), Siglecs-5-14 and -16). While “classic” Siglecs are conserved among the species, “CD33-related” Siglecs show a lower degree of conservation among species, but a higher degree of sequence similarity to each other.

Siglecs belong to the I-type family of lectins that recognize sialic acid containing glycans through their extracellular domain (ECD). Sialic acids are monosaccharides found at the termini of N-linked and O-linked glycans attached to proteins (glycoproteins) or lipids (glycolipids) on the surface of cells. Since sialic acids are found on all mammalian cells, Siglecs can help the immune system in distinguishing between self and non-self signals. Recognition of their sialylated ligands by the N-terminal variable (V)-Ig like domain, triggers cell signaling through their regulatory motifs in their cytoplasmic domains (Figure 1). For most Siglecs, these regulatory motifs are composed of immunoreceptor tyrosine-bases inhibitory motifs (ITIMs), which serve to recruit phosphatases. In the case of Siglecs-14, -15 and -16, the regulatory domains are immunoreceptor tyrosine-based activatory motifs (ITAM). Thus, Siglecs have found different ways to impart cellular responses. Their functions are shaped by the cellular distribution and ligand specificity and vary from enabling cell adhesion and/or cell signaling. Some of their diverse roles are starting to be elucidated, and have been nicely described elsewhere [3].

### 1.1. Glycan Specificities of Siglecs

**Figure 2:**
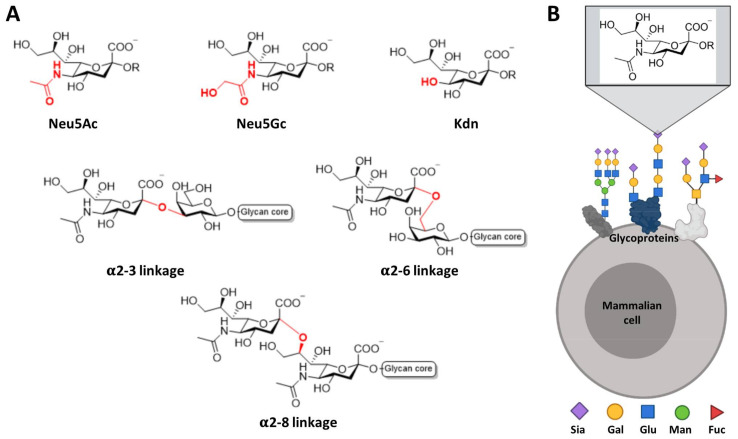
Most common sialic acids in mammals. (**A**) Chemical representation of the most common type of sialic acids in mammals and their linkage to the subterminal glycan. (**B**) Sialic acids are found at the outer most exposed non-reducing end of glycan chains on glycoproteins or glycolipids on the cell surface.

Even though all Siglecs share a common N-terminal V domain, each member presents an exclusive specificity and preferences profile towards the terminating sialic acid. Sialic acids refer to a family of nine carbon (C1-C9) sugars derived from neuraminic acid (Neu). There are more than fifty forms of naturally occurring sialic acids, all of which are derived from substituting the amine or the hydroxyl groups. From all of them, just three are mainly expressed in mammals: N-acetylneuraminic acid (Neu5Ac), N-glycolylneuraminic acid (Neu5Gc), and 2-keto-3-deoxynonic acid (Kdn) (Figure 2). However, only Neu5Ac is present in humans, since a deletion occurred in the cytidine monophosphate-N-acetylneuraminic acid hydroxylase (CMAH) enzyme gene that is responsible for converting Neu5Ac into Neu5Gc [4,5]. Some natural sialic acids bear an O-acetylation in the C9 position, which has a strong negative effect in most receptors, such as human CD22 and mouse Siglec-1 [6,7]. Regarding the C5 position of Neu5Ac, some Siglecs show different preferences toward the type of N-acyl group at that position. As an example, human and murine Sialoadhesins strongly prefer Neu5Ac over Neu5Gc; nevertheless, murine CD22 accommodates Neu5Gc better than Neu5Ac, while the human orthologue recognizes both of them [8,9].

Sialic acids can be linked to the underlying sugars by different linkages, in most of the cases by α2-3 and α2-6 type linkage to the galactose and by α2-8 to another Neu5Ac (Figure 2). In short, by summing up all forms of sialic acids, the type of linkage to the subterminal sugar, the structure of the rest of the oligosaccharide and other possible post-translational modifications (such as sulfation or N-acetylation), there are plenty of potential patterns that can be recognized with variable specificities by the Siglec receptors, which will trigger a biological response accordingly.

**Figure 3:**
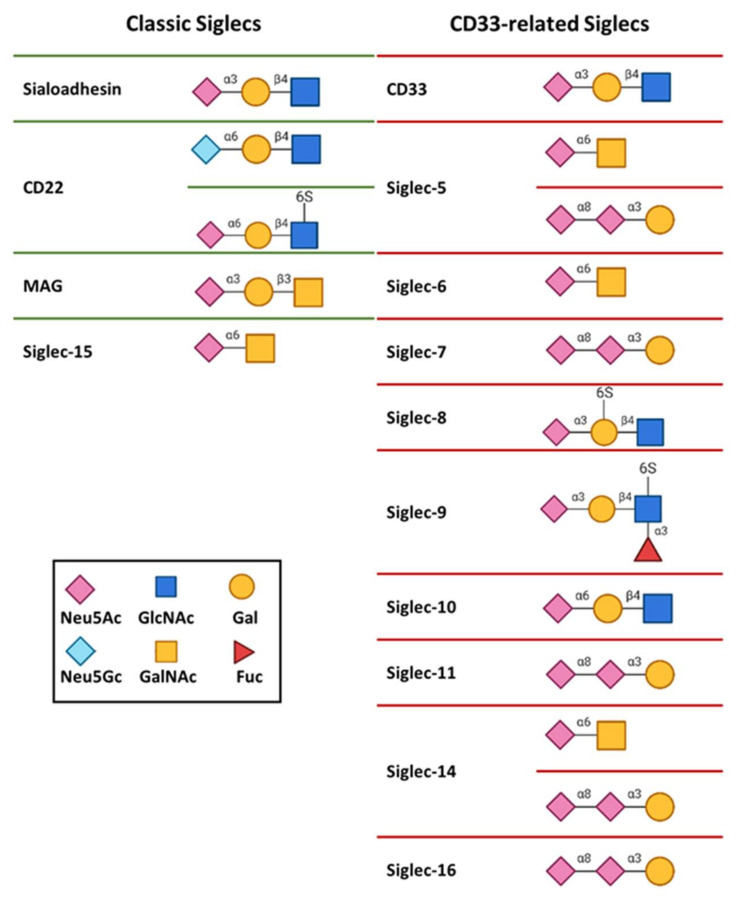
Glycan binding specificities of human Siglecs.

The binding affinities of Siglecs for isolated Neu5Acα2-6Gal and Neu5Acα2-3Gal moieties are rather low, with dissociation constants ranging from 0.1 mM to 3 mM. Despite the low binding affinity, each Siglec shows a unique specificity profile. For instance, receptor CD22 presents a strong preference for α2-6 linked sialosides, like Neu5Acα2-6Gal and Neu5Gcα2-6Gal [8,10], while Sialoadhesin leans towards α2-3 linkages [11] (Figure 3). On the other hand, Siglec-7 and Siglec-11 have marked selectivity for the Neu5Acα2-8Neu5Ac structure (Figure 3) [12,13]. The relative position of the sulfate group regarding the same sialic acid can be also a determining specificity factor. Such is the case of Siglec-8 and Siglec-9, both of which prefer Neu5Acα2-3Galβ1-4GlcNAc as ligand. However, for Siglec-8, the sulfate group at the Gal residue shows improved affinity, while Siglec-9 is more prone to bind ligand with sulfate at the glucose (Glc) moiety (Figure 3) [14,15,16].

### 1.2. Three Dimensional Structures of Siglecs

**Figure 4:**
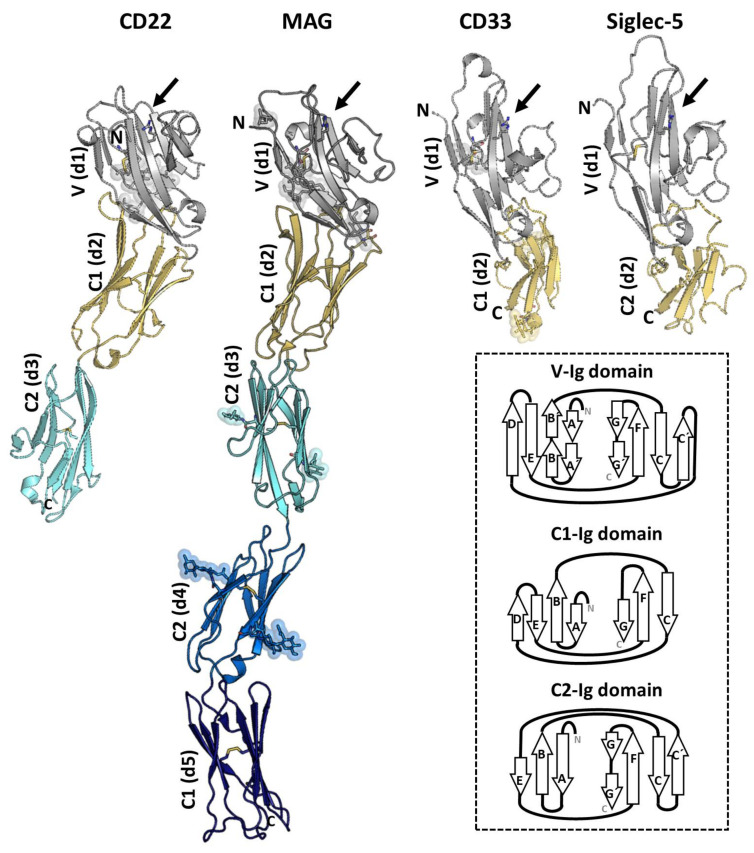
The crystal structures of CD22_d1-d3_ (PDB ID: 5VKJ), myelin-associated glycoprotein (MAG)_d1-d5_ (PDB ID: 5LFU), CD33_d1-d2_ (PDB ID: 5IHB), and Siglec-5_d1-d2_ (PDB ID: 2ZG2) in cartoon representation. Domain d1 (in grey) adopts de V-type Ig-like domain and contains the sialic acid binding pocket (indicated with an arrow) with the conserved Arg (in sticks). The N-linked glycans are represented with sticks and spheres. The disulfide bonds are also depicted with sticks. The secondary structure differences between the V- (strands A(A′)B(B′)ED and CC′FG(G′)), C1- (strands ABED and CFG) and C2- (ABE and C(C′)FG(G′) strands) type Ig folding are shown with a diagram (inside the box).

The available structural information on Siglecs by either X-Ray crystallography or NMR spectroscopy is currently limited to the ECD of the receptor. The ECDs of Siglecs contain one unique V-type Ig like domain, followed by a varying number of constant (C-) type of Ig-like domains. Structurally, Ig-like domains are composed of 70–110 amino acids, which are defined by two opposing β-sheets connected by disulfide bridges (Figure 4). To date, the 3D structures of the V-type domain (d1) of Sialoadhesin [17], Siglecs-7 [18]**,** and -8 [19] have been solved. Additionally, the full-length ECD of MAG [20] and CD33, and domains 1 to 3 (d1-d3) of CD22 (CD22_d1-d3_) [21], and d1-d2 of Siglec-5 (Siglec-5_d1-d2_)[22] have also been determined. All structures showed that the most N-terminal V-type Ig-like domains are composed by two β-sheets, A(A’)B(B’)ED and C(C′)FG(G′) linked with one intradomain disulfide bridge (Figure 4).

The analysis of the 3D structures of V-domain of Siglecs in complex with di/trisaccharides containing sialic acids has provided important clues about the recognition mode and specificity for sialic acids [17,18,19,20,21,22,23]. For most Siglecs, productive interactions with the sialoglycans are limited to the sialic acid and the adjacent Gal residues, while additional secondary binding sites have not (yet) been identified. The sialic acid binding pocket is formed by strands F and G and loops C-C′ and C′-D with a key conserved Arg residue, essential for forming the salt bridge with the negatively charged carboxyl group C1 of sialic acid. Mutation of this Arg residue causes a drastic decrease in the binding capacity of all studied Siglecs, being the mutation to the positively charged Lys less detrimental for the recognition than that to Ala. A conserved aromatic amino acid (usually a Trp) is present in all Siglec, which interacts with the glycerol side chain of the sialic acid.

**Figure 5:**
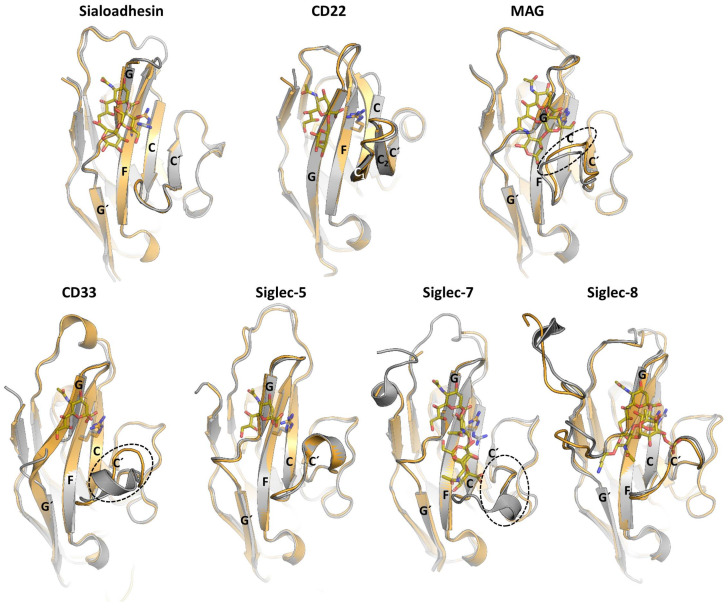
Superposition of the unliganded (grey) and liganded (orange) structures of d1 from Sialoadhesin (PDB ID: 1QFP and 1QFO), CD22 (PDB ID: 5VKJ and 5VKM), MAG (PDB ID: 5LFR and 5LF5), CD33 (PDB ID: 5IHB and 5J06), Siglec-5 (PDB ID: 2ZG2 and 2ZG3), Siglec-7 (PDB ID: 1O7S and 2HRL) and Siglec-8 (PDB ID: 2N7A and 2N7B).

Based on the available structural data, we know that the differences in loops C–C′ and strand G at the ligand-binding pocket are determinant for glycan specificity (Figure 5). The sequence variability and the conformation adopted by C-C′ loop dictates specificity for the glycan linkage and Gal moiety. Interestingly, the tip of the C-C′ loop in CD22 displays one extra β-hairpin (C1/C2) with the Tyr64, which is optimally preconfigured to extensively interact with branches of N-glycans with α2-6 linkages [21]. In Siglec-8, the edge of the CC′ loop contains the Arg56 and Gln59 side chains, to form a salt bridge and hydrogen bond with the sulfated Gal6S moiety, respectively [19]. Interestingly the binding pocket in Sialoadhesin, CD22, and Siglec-8 are preformed to accommodate the ligand (Figure 5). On the contrary, CD33, MAG, and Siglecs-7 undergo a conformational rearrangement in C-C′ loop upon ligand binding. Except for CD22, the G strand has a loop of different length inserted (Figure 5). Remarkably, the GG′ loop of Siglec-8 consists of eleven residues, which is substantially long compared with the typically five residues of most Siglecs. In Siglec-8, the long and flexible side chains of Lys120 and Gln122 on the GG′ loop interact with the GlcNAc moiety of the ligand 6′S sLex (Neu5Acα2–3[6S]Galβ1–4[Fucα1–3]GlcNAc) [19].

The 3D structures of the solved C-type Ig domains at the ECD of Siglecs can adopt either C1 (formed by strands ABED and CFG) or C2 topology (containing ABE and C(C’)FG(G’) strands) (Figure 4). The C1 or C2 Ig-like domain topology, along with differences in the length of the interdomain linkers, remarkably can change the interface between Ig domains and thus might affect the flexibility of the ECD. As shown by the crystal structure of MAG_d1-d5_ [20] and the 3D reconstruction from negative-stain EM of CD22_d1-d7_ [21], the ECD adopts a semi-rigid rod like structure that helps in projecting the ligand binding pocket at V-domain away from the cell surface (around 190 and 300 Å, respectively). Such conformation could be beneficial in exchanging binding with flexible *cis* (on the same cell surface) and *trans* (on interacting cells or molecules) ligands on the surface of the cells [20,21].

### 1.3. Targeting Siglecs for Therapeutic Purposes

Since Siglecs play important roles in the regulation of immune cells, these receptors have become important therapeutic targets [3,24,25,26]. Any therapy targeting Siglecs can exploit their ability to activate or inhibit the target cells and thus to alter their fate. Recent evidences suggest that Siglec receptors help to evade the anti-tumor immune response by engaging to cancer-associated glycans on tumor cells [27,28,29,30,31]. Upon malignant transformation, many types of cancer cells express high levels of sialic acids and cancer-associated glycans (e.g., mucins (MUC1 and MUC16), Sialyl-Tn (sTn)) on their surfaces or secrete them to the extracellular media. In breast cancer, the O-glycans of secreted mucins (e.g., MUC1 and MUC16) interact with Siglec-9 on monocytes and macrophages [32,33]. The heat stable antigen or small-cell lung carcinoma cluster 4 antigen (CD24), a heavily glycosylated glycosylphosphatidylinositol-anchored surface protein, is the ligand for Siglec-10 on tumor-associated macrophages (TAMs) and induces the inhibition of phagocytosis [34]. Similarly, many melanomas express high levels of the ganglioside GD3, which interacts with Siglec-7 on NK cells and suppresses the NK cell killing activity [13].

Additionally, the restricted expression on certain cells can be an advantage for targeted therapies. Siglec-8, for example, has garnered the attention as a target for the treatment of asthma and allergies because of its restricted expression on eosinophils and mast cells [35,36,37,38]. Siglec-15, which is mainly expressed on osteoclasts, is a potential therapeutic target for osteoporosis [39]. Another characteristic that unites most Siglecs, is that they are receptors that undergo endocytosis after binding with a ligand or antibody (Ab), and can be recycled and returned to the cell surface [35,40,41,42,43,44,45]. This feature makes Siglecs particularly attractive as therapeutic targets as it allows to carry out a ‘Trojan horse strategy’. This strategy is based on the fact that conjugating a toxin to the ligand or Ab that binds specifically to Siglec allows to deliver the toxin inside the target cell after endocytosis.

However, a critical aspect of targeting Siglecs is that we need to outcompete with natural cis and trans ligands. The local concentration of sialosides on immune cells is believed to be very high (e.g., taking into account the cell volume (210 µm^3^), glycocalyx thickness (44 µm), and the cell surface sialic acid content (2.5 µg/10^7^ lymphocytes), it was estimated over 100 mM on the surface of B cells [46]). This means that most Siglecs are masked by their interactions with nearby sialosides from the same cell (cis binders). Thus, Siglecs are believed to be organized in microdomains (e.g., nanodomains, lipid rafts, caveolae, and/or clathrin domains) at the surface of the cells [47,48]. For example, CD22 associates in highly mobile microdomains in clathrin coated pits, which are mediated by cis interactions between CD22 monomers and other cis ligands (e.g., CD45) [47].

There are numerous strategies to target Siglecs that exploit the characteristics just mentioned. The dominant strategy to target Siglecs is to use monoclonal Abs (mAbs). However, there are alternative therapies, a stand-out being the development of chemically modified glycans.

## 2. Antibody-Based Approaches to Target Siglec-Sialic Acid Axis

Anti-Siglec monoclonal Abs have emerged to modulate Siglec-sialic acid signaling. In general, the mechanism of action consists in mediating cell depletion on the targeted cell, or blocking Siglec-sialic acid interactions.

### 2.1. Anti-Siglec Antibodies for Cell Depletion

**Figure 6:**
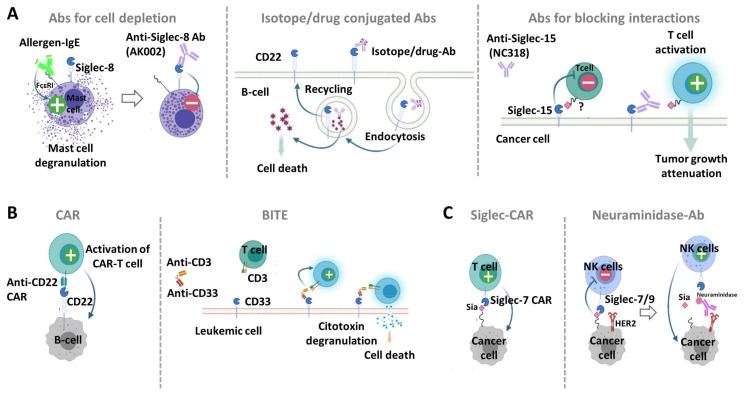
Antibody based molecules targeting Siglecs. (**A**) mAbs against Siglecs. (Left) Anti Siglec-8-Ab, such as AK002, deplete eosinophil activation and mast cell degranulation upon binding. (Middle) Isotope/drug conjugated Abs targeting CD22 on B cells are endocyted and can deliver the isotope or drug at the cytoplasm to allow cell killing. (Right) Anti-Siglec-15 Abs (e.g., NC318) can block interaction of cancer cells expressing Siglec-15 and T cells. (**B**) Targeting Siglecs with chimeric antigen receptors (CARs) and bi-specific Abs. (Left) CAR-T cells targeting CD22 have being designed to specifically target and kill malignant B cells. (Right) The bi-specific T-cell engaging Ab (BITE) targeting CD33 and CD3 on the surface of T cells is able to activate a cytotoxic response against leukemic cells without requiring the prior activation of T cells. (**C**) Targeting cancer-associated glycans of tumor cells. (Left) Siglec-7/-9 derived CAR T-cells can recognize and kill cancer cells through binding to sialylated glycans. (Right) The tumor-targeted mAbs against HER2 fused with neuraminidase, is able to degrade Siglec-7/9 ligands and restore the immune cell response.

Anti-Siglec Abs can deplete Siglec-expressing cells via recruitment of effector cells from the immune system or by direct induction of apoptosis. Many Siglecs undergo rapid internalization upon ligation by Ab, which can diminish antibody-dependent cellular cytotoxicity (ADCC) and complement-dependent cytotoxicity (CDC). This feature has also been exploited for the development of Ab drug/toxin conjugates (ADCs). Epratuzumab, a mAb targeting CD22 on B cells, relies on ADCC for antitumor activity. It has been tested clinically, and has an acceptable safety profile in patients with diffuse large B-cell lymphoma (DLBCL) and indolent non-Hodgkin lymphoma (NHL) [49,50,51]. Additionally, there are several anti-CD22 ADCs, which are internalized upon binding to CD22 and deliver chemotherapeutic molecules. The FDA has just approved inotuzumab ozogamicin (Besponsa^®^) (Pfizer Inc. and UCB S.A.) anti-CD22 ADC therapy in patients with acute lymphoblastic leukemia [52], which combines the epratuzumab anti-CD22 mAb with the cytotoxic antitumor antibiotic calicheamicin (Figure 6). Additionally, Phase I trials of moxetumomab pasudotox (Lumoxiti^TM^), an ADC which combines anti-CD22 with PE38 (a fragment of *Pseudomonas* exotoxin A), have shown promising results in hairy cell leukemia [53]. Radioimmunotherapy utilizing ⁹⁰Y-labeled epratuzumab was shown to be highly effective in patients with follicular lymphoma, generating a complete response (CR) rate of 92% and progression-free survival of more than 2 years [54]. Another Siglec that is being actively targeted in acute myeloid leukemia (AML) cells is CD33 [55,56]. CD33 is expressed at the level of hematopoietic cells, principally in circulating monocytes and dendritic cells, and is being actively targeted in acute myeloid leukemia (AML) cells [55,56]. The first FDA-approved ADC against CD33 is gentuzumab ozogamicin (Mylotarg) (Pfizer Inc.), a humanized anti-CD33 mAb covalently attached also to calicheamicin [57]. However, Mylotarg was withdrawn from the market in 2010 due to increased early deaths in newly diagnosed AML patients. In 2017, new data on the clinical efficacy and safety of Mylotarg administered on fractionated doses led to its re-approvement for newly diagnosed and relapsed AML patients [58]. Second generation ADCs that utilize biodegradable linkers and more potent toxins hold great hope for the future of CD22 or CD33-targeted therapeutics.

Siglec-8 has been proposed as therapeutic target for treating allergic and inflammatory diseases. Binding of mAbs to Siglec-8 induces apoptosis on eosinophils and inhibits IgE mediated mast cell activation (Figure 6) [38,59,60]. Additionally, anti-Siglec-8 mAbs have shown to inhibit anaphylaxis in humanized mice in a novel Siglec-8 transgenic (tg) mouse model. Herein, the human Siglec-8 transgene is constitutively expressed on murine mast cells, eosinophils, and to a lesser extent, on basophils. Currently, the humanized and non-fucosylated anti-Siglec-8 IgG1, AK002 (Allakos Inc.), is in clinical development for the treatment of allergic, inflammatory, and proliferative diseases involving eosinophils and mast cells. AK002 depletes eosinophils via ADCC and inhibits IgE-dependent MC activation. 

### 2.2. Anti-Siglec Antibodies That Block Interaction with Ligands

mAbs that block Siglec binding to ligands have been also generated to reverse the antitumor immune cell response. Recent studies have shown that Siglec-15 expresses on solid tumor cells (e.g., such as colon cancer, endometrioid cancer and thyroid cancer) and tumor associated macrophages (TAMs), and that is able to suppress T cell function [61]. Blocking Siglec-15 with a NC318 mAb (NextCure Inc.) reversed the T cell suppression, attenuating tumor growth and the ability of the tumor to metastasize to the lung level in MC-38 mice with constitutive Siglec-15 expression (Figure 6). Interestingly, the expression of Siglec-15 (which was suppressed by interferon-γ) was inversely correlated with that of PD-L1 (which was induced by interferon-γ). This finding implies that Siglec-15 may be a complementary approach for cancer patients that are refractory to anti-PD-1/PD-L1 therapies.

### 2.3. Small Peptides Derived from Anti-Siglec Abs

Although mAbs usually show high affinity (in the nanomolar range), they bind indiscriminately to healthy and malignant cells expressing the targeted Siglec receptor. One way to address the selectivity issue is to use specific anti-Siglec peptides that bind with moderate affinity (Kd in low micromolar). A short CD22 binding peptide (PV3) was recently selected from multiple peptide candidates generated from epratuzumab Fab [62], based on the crystal structure of CD22_d1-d3-_epratuzumab complex (PDB ID: 5VL3 [21]). The PV3 peptide binds with moderate affinity (Kd ∼9 μM) to CD22. This way, nanoparticles coated with PV3 are able to discern between healthy (expressing low concentrations of CD22) and malignant (expressing higher amounts of CD22) B cells. PV3-nanoparticles bound for a short time with healthy B cells, preventing the endocytosis of the nanoparticle. On the contrary, in the presence of malignant B cells, the contacting time increased because the avidity was increased. This allowed the internalization of PV3-nanoparticles and the release of the chemotherapeutic drug on malignant B cells.

## 3. Chimeric Antigen Receptors and Bispecific Engagers Directed to Siglec-Sialic Acid Axis

The redirection of T or NK cells against tumors holds much promise for the treatment of cancer. There are two main approaches for T cell redirection, which involve their genetic modifications with chimeric antigen receptors (CARs) or the use of recombinant proteins designated bispecific T-cell engagers (BiTEs).

### 3.1. Anti-Siglec Bispecific T-Cell Engagers

BiTEs are recombinant bispecific proteins that contain two linked single-chain variable fragments (scFvs) from two different Abs, one targeting a cell-surface molecule on T cells (e.g., CD3ε) and the other targeting antigens on the surface of malignant cells (Figure 6). Binding to tumor antigens and T cells simultaneously mediate T-cell responses and killing of tumor cells in a MHC independent mode. The anti-CD33/CD3 BiTE (AMG330, Amgen) was developed to recognize CD33 on AML cells and CD3 on the membrane of T cells [63]. AMG330 is able to activate a cytotoxic response against leukemic cells without requiring the prior activation of T cells or the human leukocyte antigen (HLA) system of histocompatibility. Interestingly, a number of patients with AML were found to be refractory to this type of therapy because of the presence of a single nucleotide polymorphism (SNP) (rs12459419 (C> T Ala14Val)) that leads to the loss of the CD33 V-type Ig domain [64]. In this way, the bispecific Ab JNJ-67571244 was developed to recognize the C2 domain on CD33. To date, this Ab is in phase I clinical trial for patients who have not responded to AML therapy and are at high risk of myelodysplastic syndrome. In this sense, we believe that it is relevant to analyze polymorphisms of Siglec genes and their association with disease, such as Siglec-8 and bronchial asthma [65], and Siglec-9 and lung cancer [66]. These associations will be important for the efficacy of antibody-based therapies.

### 3.2. Anti-Siglec Chimeric Antigen Receptors

The design of CARs commonly includes a single-chain variable fragment (scFv) of a given Ab specific for an antigen, an extracellular spacer and a transmembrane region as structural features, as well as signal transduction units for T cell activation (such as CD3ξ, CD28, 4-1BB, or OX40). Different studies have provided the scientific proof that the design of CARs needs to consider both the epitope position within the target antigen as well as the nature and length of the spacer region on the CAR. While membrane-distal epitopes have shown to most efficiently trigger CARs with short spacers, membrane-proximal epitopes required CARs with extended spacer domains to elicit accurate effector function [67,68,69,70,71]. Commonly used long spacer domains are the CH2-CH3 domains of IgG molecules. However, CARs containing these spacers generally bind unspecifically to Fcγ-Receptor, which contributes to an inferior in vivo cytotoxic efficacy [72]. Interestingly, in the design of novel spacers for CARs, the MAG Ig-like domain-derived spacers have shown similar attributes to IgG spacers but without unspecific off-target binding [73].

CD22 represents a validated target for CAR-T cells in B-cell malignancies (Figure 6), with potent antineoplastic effects in a phase I clinical trial enrolling patients who failed to achieve remission in the CD19 CAR-T cell therapy protocol [68,74]. A recent study found that second generation of CARs derived from anti-CD22 mAb targeting a membrane proximal C-type Ig domain (m971) has superior antileukemic activity in B-cell acute lymphoblastic leukemia (BCP-ALL), compared with those targeting membrane distal Ig domains [68]. CAR-T cells against CD33 have been also developed and tested in early phase clinical trials for the treatment of AML. A preclinical test of anti-CD33 CAR-T cells showed significant effector functions in vitro, and induced reduction of leukemia burden and prolonged survival of AML xenograft murine models. However, the anti-CD33 CAR-T cell treatment resulted in serious hematopoietic toxicity in animal models [75,76]. Therefore, novel 4th generation CARs that contain an “off switch” may be used to avoid long-term suppression of myeloid cells.

## 4. Targeting Cancer-Associated Glycans Recognized by Siglecs Using Ab-Based Approach

While Siglecs are clearly attractive targets for cancer immunotherapy, some concerns have been raised about the number of different tumor-infiltrating immune cells expressing the same Siglec, the multiple Siglecs expressed on the same cell, and the potential inhibitory action of anti-Siglec Abs. Targeting cancer-associated glycosylation patterns of tumor cells can be an effective alternative [77,78]. Recently, the efficacy of Siglec-7/9 derived CAR-T cells in eliminating tumor cells has been tested in vitro (Figure 6), in a non-histocompatibility complex molecule restricted way [79]. Siglecs-7 and -9 are expressed on NK cells, T cells, and dendritic cells and can promote immune suppression when binding to sialylated ligands on targeted cells. By genetically modifying human T cells with CARs containing Siglec-7/-9, those cells showed antitumor activity in vitro demonstrating the recognition of different cancer cell lines. Interestingly, while Siglec-7 CAR-T cells containing the extracellular Ig-like d1 and d3 (lacking d2) composed the functional building block to recognize tumor antigens, Siglec-9 CAR-T cells required d2 for its function. This finding shows that despite the high amino acid sequence identity between Siglec-7 and -9, there are structural differences on Ig-like domains that affect receptor function.

Another approach is to increase anti-tumor immunity by locally delivering sialidases or sialic acid inhibitors in the tumor microenvironment (TME). The efficacy of this technique was tested with a mAb against HER2 fused to a sialidase (Figure 6), which specifically cuts off the sialic acid ligands that are bound by Siglec-7 and Siglec-9 [80]. In vitro, the anti-HER2-sialidase Ab increased NK cell-mediated killing of HER2 positive tumor cells in breast cancer patients. On the other hand, delivery of 3F-Neu5Ac to cancer cells has shown to decrease sialic acid production by blocking the action of sialyl transferases [81].

## 5. Modified Sialic Acids Targeting Siglecs

**Figure 7:**
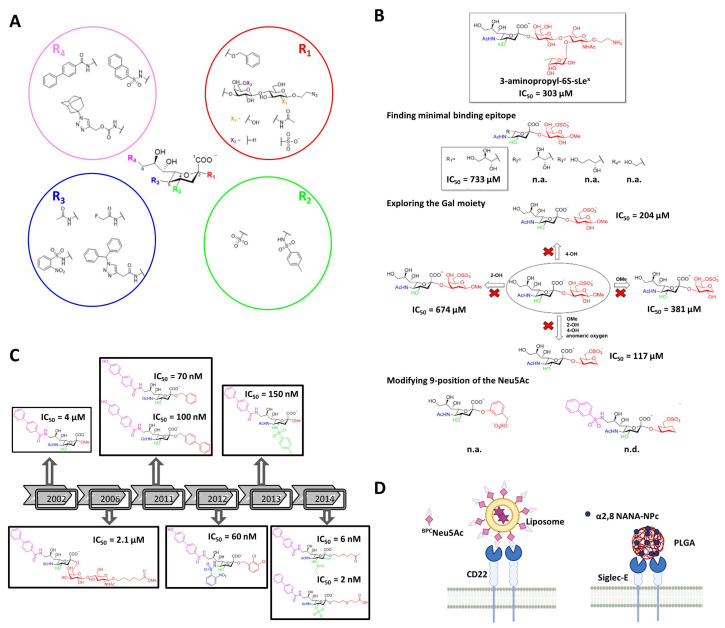
Modified glycan ligands targeting Siglecs. (**A**) General structure of sialic acid and examples of chemical substituents (R_1_–R_4_) used to generate specific and high-affinity modified glycans against Siglecs. (**B**) Development of sialic acid mimetics of high-affinity for CD22. (**C**) High-affinity ligands for Siglec-8. (**D**) Examples of nanocarriers coated with modified sialic acids.

Chemically modified glycans are drug-like compounds that mimic the structure and function of native glycans, but impart improved affinities, bioavailability and longer serum half-lives [82]. Siglec targeting modified glycans are based on synthetically modified sialic acid scaffolds. These compounds need increased potency for binding at the binding groove masked by the endogenous *cis* glycans on the target cells. Except for the carboxylic C1 position, which is essential for binding to Siglecs, the rest of the scaffold ranging from C2 to C9 can be potentially modified (Figure 7).

The first pioneers developing new class of high affinity sialic acid analogues were Kelm et al., with the purpose of addressing the role of the ligand binding domain of CD22 [83]. Most of the variables introduced at positions C5 and C9 on Neu5Ac had negative effect on the binding. However, some substituents such as an -NH_2_ group in C9 (9-NH_2_-Neu5Ac/Me) and a fluoroacetate group at C5 (Neu5FAc/Me) enhanced the affinity considerably. The improvements were due to the extra hydrogen bonding and lipophilic interactions between the synthetic ligand and CD22. These observations opened the door to the design and synthesis of new unnatural glycans against CD22 [84,85,86]. Years later, Kelm et al. exploited the hydrophobic pocket next to the position C9 on CD22, to synthesize a biphenyl (BPC-Neu5Ac) substituted ligand with a 244-fold increase potency (IC_50_ = 4 µM) and improved selectivity (Figure 7) [85]. Thereafter, two different strategies have been followed regarding the choice of underlying structure at the C2 position. On the one hand, there is the strategy of adding the preferred subterminal glycan (Neu5Acα2-6Galβ1-4GlcNAc) at the C2 position and combining it with further modifications at C9 (e.g., BPC) [87]. However, the affinity IC_50_ (2.1 µM) was not significantly improved respect to the previous BPC-Neu5Ac. On the other hand, substituting the C2 position with hydrophobic groups (e.g., phenyl, biphenyl) has skyrocketed the potency (IC_50_) to the nanomolar range (100 nM) [88,89].

As can be concluded from the examples showed above, modifications at various positions on the sialic acid could improve the binding affinity with CD22. Based on this, Mesch et al. combined multiple modifications simultaneously at C2, C5 and C9 positions [90]. The best ligand combined an o-nosyl group at C2, a α2,3-dicholorobenzyl group at C5, a 4-(4-hydroxy) biphenyl substituent at C9 and exhibited a KD of 60 nM (Figure 7). However, the pharmacokinetic properties of this compound were not optimal for its oral administration. Later on, replacing the carboxylate group with a bioisostere improved the half-life of the compound compared to natural sialosides and showed good binding to plasma proteins [90]. Afterwards, Kelm et al. reported for the first time the modification of C4, together with C9 [91]. A nitrophenylcarboxamido group was introduced at C4, which lead to a 15-fold increase in the binding. This showed a synergistic effect together with a BPC substitution at C9, with an overall of 9100-fold increase. Saturation transfer difference (STD) NMR indicated that C9 and C4 modified ligands bound to the same binding pocket as the natural ligand and that these synthetic substituents interact with the binding groove. Thereafter, Prescher et al. synthetized mimetics with modifications at three positions simultaneously (C3, C4, and C9), and compared the results with those obtained for a molecules with four simultaneous (C2, C3, C4, and C9) modifications [92]. Interestingly, while the mimetic with four variations presented 4 nM affinity, that with three decorations exhibited 2 nM affinity.

Recently, the development of a potent mimetic for Siglec-8 using the classical structure activity relationship (SAR) process, and based on the NMR structure of Siglec-8 V domain with the ligand 3-aminopropyl 6′-sulfo-sLe^X^ [19], was published by Kroezen et al. [93]. The 3D structure of the complex revealed that the L-Fucose and D-Glc*N*Ac moieties do not engage any interactions with the protein surface, indicating they could be removed in order to find the minimal binding epitope. In addition, the glycerol side chain of the Neu5Ac results to be important due to the extensive hydrogen bonding network that is involved in, specially the 8-OH that stabilizes the bioactive conformation of the sialic acid. Thereafter, bioisosteres of the carboxylate and sulfate group were explored, turning out that no replacement was needed since these were the most active ones. According to the obtained NMR structure of the complex, the 2- and 4-OH of the Gal moiety do not notably contribute to the binding. Furthermore, the anomeric substituent cannot stablish any interaction since it points away from the protein surface. Therefore, a set of derivatives were synthetized where these three substituents were consecutively removed and tested. The replacement of the Gal by a cyclohexane and maintaining the sulfate at position 6 improved the IC_50_ to 117 μM. Finally, modifications were tried in the 9 position of the Neu5Ac moiety, where the naphthyl sulfonamide substitution skyrocketed the affinity to 15 μM (Figure 7). Eventually, ITC studies where carried out with these compounds, concluding that a compelling improvement in ΔS° (difference in entropy) was achieved, which resulted in a 10-fold increment in the residence time of the final compound.

The mentioned modified sialic acids were facilitated by classical synthetic structure activity relationship (SAR) studies, which are rather slow and time consuming. In this context, a new high-throughput strategy has been developed by using the copper(I)-catalyzed azide-alkyne cycloaddition (CuAAC) reaction, which eases the synthesis and screening of sialic acid analogue libraries. The CuAAC reaction has allowed to introduce a 1,2,3-triazole scaffold plus any substituent at the desired position [94]. The analogues are printed as a microarray on glass slides, where can be tested with fluorescently labelled recombinant Siglecs fused to fragment crystallizable (Fc) region of Ab. Using such approach Rillahan et al. identified ligands for CD33 and Siglecs-5, -7, -9, and -10 in the absence of structural information for the majority of the family members [94]. Even though no IC50 values were measured, the approach allowed to compare the relative affinities towards different Siglec members. Important information about selectivity was derived, indicating that the most potent ligands were not necessarily the most selective ones, such as for Siglec-5, where its most potent ligand also presented high affinity for Siglec-9.

### 5.1. Nanocarriers Decorated with Modified Glycans

Even though high affinity ligands for almost each Siglec have been developed, most of the ligands are in the low micromolar range affinities [95]. Moreover, high avidity binding and clustering of the receptor is required in order to outcompete natural ligands. A promising alternative is the use of multivalent display of mimetics onto a nanoparticles, liposomes or polymers [96] (Figure 7). Bio-orthogonal synthesis is enabling the presentation of modified sialic acids on nanoparticles, polymers, and living cells [97,98].

Liposomes coated with BPC-Neu5Ac loaded with cytotoxic cargo doxorubicin can target CD22 expressing B-cell lymphoma cells [99]. Since CD22 is an endocytic receptor, liposomes are rapidly endocytosed, glycans released in the acidic endosome, and accumulated inside the cell over time. On the contrary, Abs are not released in the endosome, and recycle back with CD22 to the cell surface. The BPC-Neu5Ac-liposomes were able to prolong the lives of tumor-bearing animals compared with the nontargeted liposomal ones. Apart from delivering cargo, glycan decorated nanoparticles can be used for stimulating Siglec signaling. For instance, Macauley et al. presented high affinity binding ligands for CD22 on liposomal surfaces that were able to inhibit B cells signaling, conducting to cellular apoptosis and antigen-specific tolerance induction [100]. Liposomes containing a synthetic 9-*N-*sulfonyl sialosides analogue targeting Siglec-8 have also shown strong in vitro binding, uptake and selectivity to Siglec-8 expressing cells [101].

Poly(lactic-co-glycolic acid nanoparticles (PLGA) decorated with di(a2→8) N-acetylneuraminic acid (a2,8 NANA-NP) have been shown to block the production of lipopolysaccharide-induced inflammatory cytokines by macrophages in a Siglec-E (functional orthologue of human Siglec-9)–dependent manner [102]. The nanoparticles were also therapeutically beneficial in vivo in both systemic and pulmonary murine models replicating inflammatory features of sepsis and acute respiratory distress syndrome (ARDS). Moreover, these nanoparticles had an anti-inflammatory effect on human monocytes and macrophages in vitro and in a human ex vivo lung perfusion (EVLP) model of lung injury.

### 5.2. Multivalent Modified Glycans

Paucivalent ligands (di-, tri- or tetravalent) represent an alternative approach to nanoparticles. In this sense, branched N-linked glycans can serve as scaffold by itself [103]. N-Linked glycans chemoenzymatically functionalized with BPC-Neu5Ac or MPB-Neu5Ac have been tested against CD22 [104]. Interestingly, di- and tri-branched ligands based on natural N-linked glycan scaffolds provided the biological spacing needed to increase affinity for CD22 from micromolar to low nanomolar/high picomolar, allowing competition with cis-ligands on B cells. Moreover, these ligands were conjugated with toxins and endocyted by Daudi B lymphoma cells expressing CD22, promoting cell killing.

## 6. Conclusions

In recent years much has been learned about the role of Siglec-sialic acid axis in the immune modulation and their implication on diseases, but still little is known about the natural ligands. Advances in biochemical tools and disease models have paved the way for different therapeutic molecules that target Siglecs to modulate the immune cells. The number of clinical trials targeting Siglecs (mostly CD22 and CD33) continue to increase, especially thanks to the development of bi-specific Abs and CARs. However, therapeutic Abs present some functional limitations such as inadequate pharmacokinetics and tissue accessibility, apart from harmful interactions with the immune system that can cause serious side effects. In certain applications, glycan ligands have an advantage over Abs, such as their ability to dissociate from their target once endocytosed. Due to this, in the last decade many efforts have been made for finding specific modified glycans for Siglecs. As our knowledge of the physiological functions of Siglecs receptors and the nature of sialylated ligands continues to expand, so will the opportunities to modulate Siglec-sialic axis.
